# Factors affecting implementation of a National Clinical Programme for self-harm in hospital emergency departments: a qualitative study

**DOI:** 10.1136/bmjqs-2024-017415

**Published:** 2024-10-08

**Authors:** Selena O'Connell, Grace Cully, Sheena McHugh, Margaret Maxwell, Anne Jeffers, Katerina Kavalidou, Sally Lovejoy, Rhona Jennings, Vincent Russell, Ella Arensman, Eve Griffin

**Affiliations:** 1 School of Public Health, University College Cork, Cork, Ireland; 2 National Suicide Research Foundation, Cork, Cork, Ireland; 3 Nursing, Midwifery and Allied Health Professions Research Unit, University of Stirling, Stirling, UK; 4 National Clinical Programme for Self-harm and Suicide-related Ideation, Health Service Executive, Dublin, Ireland; 5 Department of Psychiatry, Royal College of Surgeons in Ireland Faculty of Medicine and Health Sciences, Dublin, Ireland; 6 Australian Institute for Suicide Research and Prevention, School of Applied Psychology, Griffith University, Brisbane, Queensland, Australia

**Keywords:** Qualitative research, Emergency department, Mental health, Implementation science

## Abstract

**Background:**

A substantial number of people experiencing self-harm or suicidal ideation present to hospital emergency departments (EDs). In 2014, a National Clinical Programme was introduced in EDs in Ireland to standardise care provision. Internationally, there has been limited research on the factors affecting the implementation of care for people who present with mental health crises in EDs.

**Methods:**

This qualitative study examined factors influencing the implementation of the National Clinical Programme for Self-harm and Suicide-related Ideation in 15 hospitals in Ireland from early (2015–2017) through to later implementation (2019–2022). Semi-structured interviews were conducted with staff involved in programme delivery, with the topic guide and thematic analysis informed by the Consolidated Framework for Implementation Research.

**Results:**

A total of 30 participants completed interviews: nurse specialists (n=16), consultant psychiatrists (n=6), nursing managers (n=2), emergency medicine staff (n=2) and members of the national programme team (n=4). Enablers of implementation included the introduction of national, standardised guidance for EDs; implementation strategies led by the national programme team; and training and support for nurse specialists. The following inner-setting factors were perceived as barriers to implementation in some hospitals: limited access to a designated assessment room, delayed access to clinical input and poor collaboration with ED staff. Overall, these barriers dissipated over time, owing to implementation strategies at national and local levels. The varied availability of aftercare impacted providers’ ability to deliver the programme and the adaptability of programme delivery had a mixed impact across hospitals.

**Conclusions:**

The perceived value of the programme and national leadership helped to advance implementation. Strategies related to ongoing training and education, developing stakeholder interrelationships and evaluation and monitoring have helped address implementation barriers and promote continued sustainment of the programme. Continued efforts are needed to support nurse specialists delivering the programme and foster partnerships with community providers to improve the transition to aftercare.

**WHAT IS ALREADY KNOWN ON THIS TOPIC**

**WHAT THIS STUDY ADDS**

- This study describes barriers and enablers of the implementation and sustainment of a national programme for self-harm and suicidal ideation. Enablers spanned a range of levels from national leadership and providers’ positive perceptions of the programme through to local resources and collaborative relationships with ED and aftercare providers.

**HOW THIS STUDY MIGHT AFFECT RESEARCH, PRACTICE OR POLICY**

- The study identified factors affecting the implementation of the programme over time and strategies used to address barriers, providing actionable learning for national, ED-based programmes.

## Introduction

 The emergency department (ED) has been identified as a key setting for intervention with persons who experience self-harm or suicidal crisis.^1^ Studies indicate that 30–50% of those who die by suicide have attended a hospital ED within the previous year.^1^,^5^ People who present to the hospital with self-harm are at increased risk of dying by suicide,^6 7^ while those presenting with suicidal ideation have been found to be at increased risk for subsequent self-harm and suicide.^8 9^

Guidelines have been developed that identify best practices for responding to people presenting to healthcare services with self-harm. The National Institute for Health and Care Excellence (NICE) has produced clinical guidelines on assessing and managing self-harm for use within the National Health Service (NHS) in the UK,^10^,^12^ which have undergone updates since the original guidelines in 2004. In Ireland, an evidence-based model of care, the National Clinical Programme for Self-harm and Suicide-related Ideation (NCPSHI),^13^ was launched in 2014. The NCPSHI is a national health system-level programme that involves a mental healthcare pathway within the ED environment. It is primarily delivered by trained clinical nurse specialists (CNSs) funded through the NCPSHI with a remit specific to self-harm/suicidal ideation in the acute hospital who work in collaboration with ED staff. The programme comprised key components of care that reflect the NICE guidelines including an empathic, validating response, a timely and comprehensive biopsychosocial assessment, involvement of family/carers, development of an emergency care plan (similar to safety planning), follow-up with the person after leaving the ED and bridging them to their aftercare, that is, follow-on care by the community or primary care providers.

However, research has highlighted variation in the implementation of these guidelines and recommended care components. A survey conducted among healthcare professionals working across primary, secondary and community care in the UK found that the NICE guidelines^10^,^12^ were implemented in just under 50% of encounters.^14^ A study of Irish data from 2017 to 2018 found that, of those discharged from the ED following a self-harm presentation, on average 77% had received a psychosocial assessment.^15^ However, the provision of psychosocial assessment ranged from 17% to 97% across hospitals, with similar variation reported in the UK.^15 16^ While large-scale data on the implementation of other care components is limited, their delivery may be more varied than psychosocial assessment. A survey of providers across hospitals in the USA found that the implementation of elements of safety planning^17^ was typically less frequent than the implementation of assessment.^18^

In previous work, we examined the impact of the introduction of the NCPSHI on patient and care outcomes.^19^ The 15 studied hospitals had varied liaison psychiatry services in place prior to implementation, that is, psychiatry services operating at the intersection of mental and physical healthcare, of which a key focus is the ED.^20^ Our study found that the implementation of the programme was associated with improvements in the provision of care across hospitals. The greatest improvements were in hospitals without liaison psychiatry staff prior to implementation, where there was a reduced risk of non-assessment and increased referrals to mental healthcare for those presenting with self-harm.^19^ However, our understanding of the factors contributing to the observed outcomes remains limited.

In the broader literature, there has been little research on the contextual factors influencing the implementation of guidance for self-harm or suicidal ideation in healthcare settings.^21^ Previous studies on experiences of providing care for self-harm or suicidal crisis in the ED have focused on care predominantly provided by ED providers in the absence of dedicated mental health staff.^22 23^ Furthermore, there has been limited research on the sustainment of innovations, which is a key concern to ensure continued implementation of best practices in the long term.^24^ The aim of this study was to explore the factors influencing the implementation of the NCPSHI over time and to identify the strategies used to advance the implementation of the programme.

## Methods

### Study design

A qualitative study design was used to explore the implementation determinants of the NCPSHI in EDs across Ireland, using the revised Consolidated Framework for Implementation Research V.2.0 (CFIR).^25 26^ As our aim was to understand the barriers and enablers of implementation, we used a determinant framework rather than a process model or evaluation framework.^27^ This is the second phase of a larger project that used a natural experiment design to examine the impact of the NCPSHI on patient outcomes and provision of care.^19 28^ Consolidated criteria for Reporting Qualitative research (COREQ) were used.

### Setting

The NCPSHI, henceforth described as the programme, was developed in 2014 for adults presenting with self-harm to hospital EDs^13^ and later expanded to cover self-harm and suicidal ideation across community and hospital environments.^29^ Further description of the model and infrastructure is available in the protocol paper.^28^ While the NCPSHI targeted all EDs across 26 public hospitals in Ireland, implementation was staggered across hospitals. Following on from the analysis of the impact evaluation,^19^ the same subset of hospitals (n=15) that were implementing the programme by January 2015 were included in this study^19^

### Participants and recruitment

Members of the national programme team and staff from the included hospitals were eligible to participate including CNSs, clinical leads (consultant psychiatrists at the hospital level), nursing managers and emergency medicine staff. Individuals who currently or previously held the described roles were eligible, provided they had more than 6 months’ experience working on the programme. An email inviting participation in the study was circulated to all potential participants. A purposeful sampling strategy was used to guide follow-up emails. This was done to ensure representation across different types of hospitals (according to pre-existing services prior to the introduction of the programme) and to ensure representation from CNSs (as the primary implementors of the programme) and to a lesser extent, consultants, nursing management and ED representatives. The purposeful sampling strategy also prioritised participants who had been involved in the early implementation of the programme. Across the national programme team and NCPSHI network staff, 65 people were invited to participate with a total of 30 participants taking part in once-off interviews.

### Data collection

Semi-structured individual interviews were conducted with topic guides informed by CFIR V.2.0^25 26^ and NCPSHI programme documentation (see topic guide for clinical staff in online supplemental file 1). CFIR is an implementation determinant framework that guides the user to explore a range of factors that may influence implementation across five domains: innovation, inner setting, outer setting, individuals involved in implementation and implementation process.^25 26^ Given that we were seeking experiences of implementation over a long period of time (2015–2022), we structured our questions in two sections: first, experiences of early years of implementation from 2015 to 2017 (where applicable), and second, experiences of implementation from 2019 onwards. These timeframes were chosen to help orient participants to describe early experiences of implementation and to draw comparisons to more recent implementation when it would be expected that the programme would be more embedded in routine care, anchored as the year prior to the COVID-19 pandemic. The analysis reported in this paper excludes experiences of implementing the programme during the initial months of the COVID-19 pandemic. This analytical choice was made because we wanted to ensure we captured the primary factors affecting typical implementation of this national multi-level programme, while the initial months of the pandemic represented a unique crisis time. The interviews were tailored to the participant’s role and covered perceptions of the core components of the programme and barriers and enablers of implementing the core components, with prompts to explore factors across the CFIR domains.

Interviews were conducted by postdoctoral researchers SOC and GC who were independent of the healthcare organisation delivering the programme. The interviewers had prior professional connections with some participants. Interviewers had previous experience conducting qualitative interviews and took brief notes during interviews on topics for follow-up questioning. Interviewers engaged in reflection on the interview content and process following the interviews through research team discussion.

Most interviews were conducted via video call (n=27; audio-only was used for sections of interviews where challenges emerged with video/connectivity) with the remainder conducted in person (n=3). Interviews lasted approximately 48 min (median, IQR=19) and were conducted between November 2022 and April 2023.

### Data analysis

Interviews were audio recorded, transcribed and pseudonymised. Thematic analysis was used, drawing on a codebook approach.^30 31^ Coding was primarily deductive according to the CFIR V.2.0 constructs, with operational definitions generated for this study.^25 26^ Additional codes were added inductively as needed to capture the data. All interviews were coded by SOC and 20% (n=6) were independently coded by GC. The codebook and operational definitions of constructs were refined following comparison and discussion among the two coders and then applied to all interviews following consensus. NVivo (Release 1.7) software was used to manage data and coding.

Themes were developed iteratively. Initially, influential CFIR constructs within each domain were identified based on the frequency of discussion across participants or their emphasis as influential factors within participants’ accounts. Following the presentation and discussion of findings at meetings of the research team and working group, inductive themes were developed to capture the factors influencing implementation, in some cases drawing together constructs across CFIR domains to tell the story of implementation (see online supplemental file 2 for a mapping of influential CFIR constructs to developed themes). Implementation strategies described by participants were coded inductively through the thematic analysis and subsequently mapped to the Expert Recommendations for Implementing Change (ERIC).^32 33^ Final themes were reviewed by the research team and working group and presented to the national programme team and clinical staff of the programme for feedback.

## Findings

Thirty participants represented all 15 hospitals in the study. Most participants were CNSs (n=16), followed by clinical leads (n=6), members of the national programme team (n=4), nursing management representatives (n=2) and emergency medicine representatives (n=2). Participants were a median of 7.5 (IQR=6) years in their role, with two-thirds of participants in their role by 2017 (n=20; 66.7%).

**Figure 1:**
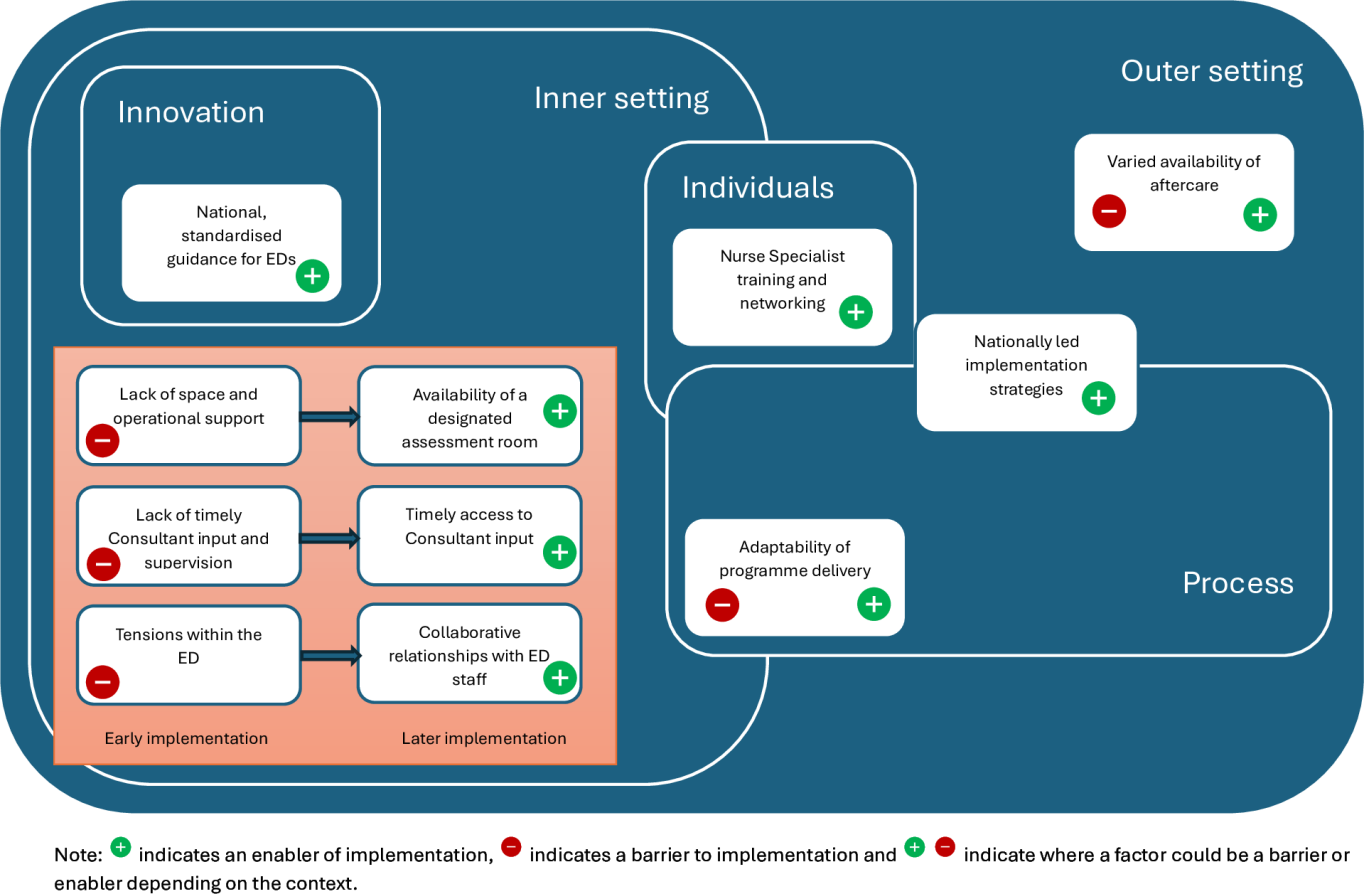
Factors affecting implementation of the National Clinical Programme for Self-harm and Suicide-related Ideation within Consolidated Framework for Implementation Research domains. EDs, emergency departments.

We developed eight themes to describe the factors affecting implementation. Themes reflected different CFIR constructs, sometimes cutting across domains (see online supplemental file 2). We have presented themes within the CFIR domain they most closely correspond to and illustrate the overlap in figure 1. In summary, the innovation, in this case the NCPSHI, provided national standardised guidance for EDs, enabling implementation. Within the inner hospital setting, limited operational resources, difficulties accessing consultant clinical input and tensions within the ED acted as barriers in the early years. Increases in these resources and the development of relationships over time enabled implementation in later years (see figure 1). Nurse specialist training and networking enabled CNSs to deliver the NCPSHI. Varied availability of aftercare within the community, that is, the outer setting, acted as a barrier to implementation. In terms of the implementation process, the adaptability of programme delivery had the potential to be an enabler or a barrier depending on the context while nationally led implementation strategies helped to address barriers and enabled implementation over time.

### Innovation

#### National, standardised guidance for EDs

Participants predominantly perceived the programme and its approach to providing care as valuable, which fostered support for implementation. Participants frequently highlighted that the programme provided a standardised approach by identifying key components of care, as well as by allocating dedicated CNSs based on the number of relevant presentations to each hospital. The model of care and associated standard operating procedures offered a consistent approach across EDs or a ‘tightening up’ of practices (table 1, quote A). Providers also identified the advantage that the programme had a ‘clear beginning and end’ within the ED, with steps from assessment through to care planning and follow-up phone call. However, there was an initial delay in the release of the formal programme documentation, which meant that CNSs often commenced positions without clear guidance, and some described the role initially as ‘daunting’.

**Table 1:** Themes and illustrative quotations organised by CFIR domains

CFIR domain	Theme	Quotation
Innovation	National, standardised guidance for EDs	(A) “When the programme came in, I suppose it just gave more specific guidelines as to the biopsychosocial assessment, the 24hr phone calls, the next care, the GP letter and the next of kin involvement and stuff like that.” (CNS, ID103)
Inner setting	Designated space and operational support	(B) “We’ve used the (audit) to bring about change or upgrades to our assessment room in the A&E Department to make sure that that’s fully compliant, so we like the fact that the National Programme come looking annually or every second year to us around the room, because that’s useful to bring to the hospital management to say if we need to upgrade, and the hospital are very supportive always of that.” (Clinical lead, ID105) (C) “If there was an issue, we would always get support (from nursing manager), but I would always have to go looking for the support, as opposed to there being a regular check-in.” (CNS, ID118)
	Timely access to clinical input and supervision	(D) “There’s always a consultant on hand (…) we can just discuss a patient, we can ring up and say, “Look, this is the presentation, this is what I was thinking as a plan for them going forward,” and they might add something to it. Or they might say, “Look, I think I need to see that patient, from what you’re saying, my sense is…” and they’d come down and review the patient. So yeah, we are very well supported clinically.” (CNS, ID108)
	Navigating tensions in the ED through collaborative relationships	(E) “I just think nurture the relationship that you have with the emergency department really. You want something that’s mutually respectful. And I think that’s why we’ve been so fortunate in how we’ve rolled out the programme here because we’re a face. I think you have to make your presence known, encourage them, ED to contact you. If they are unsure about something, contact you.” (CNS, ID106)
Individuals	Nurse specialist training and networking	(F) “I think the programme was in its infancy then at that point so lots has happened and developed since that, but it was definitely talking to other people, finding out what they were doing in other areas that I figured out, and also there were national meetings which were fantastic because you got a feel for what was going on around the country.” (CNS, ID109)
Outer setting	Varied availability of aftercare	(G) “I would phone to find out when was their appointment with the community mental health team, and it might be four weeks’ time, five weeks’ time. So, as a self-harm nurse, also we were supposed to provide brief follow-up. And that’s not brief follow-up.” (CNS, ID121)
Implementation process	Adaptability of programme delivery	(H) “There was no real roll-out in the sense of planning for organisational structure, and the governance for the roles, and how the roles would interface between the mental health services and the medical services.” (CNS, ID123) (I) “I would still pick up bits of liaison work, to help out my colleagues, and I find that great, because it keeps you fresh, and it keeps you from getting a bit bogged down in self-harm all the time.” (CNS, ID118)
	Nationally led implementation strategies	(J) “A lot of it depends on the working relationships that the clinical programme and the clinicians on the clinical programme develop with the services and service providers and clinical leads of those services.” (Programme team member, ID130)

CFIR, Consolidated Framework for Implementation Research; CNS, clinical nurse specialist ; ED, emergency department.

### Inner setting

#### Designated space and operational support

Securing an appropriate assessment room was a prominent challenge in the early years across hospitals and reflected the competing priorities within the hospital environment. CNSs reported how they tried to work around the lack of a designated private, safe space, but this resulted in extra demands on CNS time and delays in care. An audit process introduced by the national programme team was viewed to be helpful in identifying resource gaps related to the assessment room and in advocating for these resources by CNSs, clinical leads and nursing managers (table 1, quote B). Access to an appropriate assessment room improved over time for most hospitals but was still being navigated by some.

Both CNSs and clinical leads highlighted the challenge of adequate time for CNSs to conduct all the work related to their role, and balancing core clinical work alongside administrative, educational and data input responsibilities. Some participants suggested a need for protected time, more clerical resources, or improved IT systems to address these challenges and improve systems of handover. Local nursing managers were responsible for providing managerial supervision to CNSs which included support in terms of acquiring resources and materials. Nursing managers were limited in the time available to provide this support to the wide breadth of their role and competing priorities (table 1, quote C).

#### Timely access to clinical input and supervision

It is recommended practice within the programme for CNSs to discuss each case with a consultant to agree the care plan. Consultant support was instrumental to make collaborative decisions regarding patient care planning and for CNSs to feel supported in their role and not overburdened by ‘holding the risk’ (table 1, quote D). Participants described how this process is now well established across hospitals, but some struggled to get consultant support in the early years, particularly in settings where there was no existing consultant-led liaison psychiatry team.

CNSs valued clinical supervision—a regular meeting for reflection and developing competence for the CNSs—but the availability of supervision varied across hospitals and depended on the existence of a liaison psychiatry team. Some CNSs reported having no supervision for long periods of programme implementation. The support of other team members such as other CNSs and psychiatry trainees was also valued. This included checking in with each other, talking through cases, the opportunity to ‘bounce ideas’ off each other and sharing workload to prevent any one member from becoming overburdened.

#### Navigating tensions in the ED through collaborative relationships

Participants identified that ED and hospital staff were often supportive of the programme given the growing number of presentations of self-harm and suicidal ideation and the risk profile of the patient group. However, the programme staff also identified some resistance to change. There were challenges in implementing new practices around parallel assessment and management of both psychosocial and medical needs of patients. Additionally, the length of time needed to carry out programme components were at odds with the fast pace of the ED. Some ED staff were perceived to be apprehensive around handling presentations related to self-harm or suicidal ideation, to lack awareness or hold stigmatising beliefs, particularly in the early years. One participant highlighted that some ED staff did not “feel comfortable or skilled in addressing those questions with people, (…) talking about problems that people are often secretive about and ashamed of” (CNS, ID123).

The programme recommended a train-the-trainer model, where CNSs were trained to provide education sessions to ED staff and other healthcare professionals. Following attempts at providing training in the early years, CNSs identified that the recommended 3-hour trainings were not feasible. The strategy was later tailored to deliver shorter sessions (eg, 30 min) that slotted in with existing education timetables and the availability of ED staff. Participants felt these trainings were helpful in opening up the conversation, developing relationships and creating shifts in knowledge and attitudes.

CNSs and clinical leads often reported actively working to develop and strengthen relationships with the ED staff, which improved over time and were seen as paramount to the implementation of the programme. Participants described how these relationships were strengthened by the visibility and timely response of the CNSs in the ED, formal meetings and informal conversations to support ED staff (table 1, quote E).

### Individuals

#### Nurse specialist training and networking

Participants across all roles identified that CNSs have become highly skilled in assessing and care planning related to self-harm and suicidal ideation. CNSs themselves described developing confidence in their skills over time, as this role was notably different from previous roles in the ward environment for many, including greater autonomy of work and greater ‘burden of responsibility’. From early years, training days helped CNSs build confidence in their role and challenged beliefs and attitudes about people who engage in self-harm. CNSs and clinical leads also valued learning from other sites. This was mostly through ongoing training meetings, which brought programme staff together and provided opportunities for shared learning across hospitals. Some CNSs visited other sites to share learning, particularly in the early years (table 1, quote F).

Participants identified the high potential for ‘burnout’ among CNSs due to the challenging nature of crisis situations within the role along with time pressures and limited clinical input and supervision in some hospitals. The risk of burnout and difficulties in career progression were identified as contributing to the turnover of CNSs, with CNSs reporting trouble progressing to more senior roles. Participants highlighted how the motivation and engagement of the CNSs was critical to the continued provision of the programme and its sustainment over time, acknowledging the need to support CNSs in their role.

### Outer setting

#### Varied availability of aftercare

The programme outlined that CNSs would make referrals/signpost to appropriate aftercare, follow-up with the person who presented and bridge them to their next care provider. There was variability in the extent to which timely aftercare was available in each catchment through community providers and teams. Delays in aftercare increased the time demands of the CNS (table 1, quote G).

Some community teams were described as unable to provide timely care due to being extremely busy, but programme staff also reported an unwillingness to accept a ‘nurse referral’ in some teams, which resulted in ‘relationship breakdown’. Community teams were also not available out-of-hours or at weekends, which hindered CNSs from providing aftercare appointment details during those times. Communication and relationships with aftercare providers and the range of services available for aftercare developed in some regions over time. Links with general practitioners were generally perceived as positive, with CNSs liaising with them for information and follow-up.

### Implementation process

#### Adaptability of programme delivery

From the outset of implementation, there was some flexibility as to how the programme would be delivered in each hospital. Some hospitals shared the workload of liaison psychiatry across the team, whereas in others, CNSs were employed specifically to work within the remit of the programme while other colleagues were designated to cover other liaison work. While this adaptability worked well in many hospitals, it was challenging in a few and taken as evidence of a lack of planning or consideration of how the programme would fit with pre-existing services (table 1, quote H). For some CNSs, the variety of work associated with the broader liaison psychiatry role was seen as being integral to job satisfaction and engagement (table 1, quote I).

In the early years, there was reported variation in the hours covered by CNSs across hospitals, ranging from 24 hours/7 day cover to 8 hour/5 day cover. In many hospitals, the hours covered by CNSs have expanded over time or are in the process of expanding. In most hospitals, psychiatry trainees, also known as non-consultant hospital doctors, deliver the programme out-of-hours. However, the ability of psychiatry trainees to deliver the full programme components and handover cases to the CNSs was affected by time pressures with an increased number of presentations at night and on weekends and the rotation of psychiatry trainees between settings every 6 months, which contributed to inexperience with the programme and the need to regularly build new relationships and provide education.

#### Nationally led implementation strategies

At the outset, national funding enabled the recruitment of CNSs to deliver the programme across all hospital EDs. The support of the national programme team was perceived positively by most participants delivering the programme. Some programme team members felt that they had been limited in the extent to which they can influence operations or require cooperation from staff at the hospital level, relying on relationships and goodwill to implement the programme (table 1, quote J).

A number of implementation strategies were coordinated by the national programme team, which were perceived to advance implementation (table 2). As recommended in the model of care, the programme team placed an emphasis on monitoring systems from the outset of the programme, creating a data collection template completed by CNSs. Having this data enabled implementation by helping to identify gaps in services and advocate for resources. In later years, the national programme team employed further implementation strategies to address challenges, including audits of programme resources and outcomes, site visits and meetings with hospital management and the development of national working groups to guide implementation.

**Table 2:** Implementation strategies used to implement the NCPSHI in hospital EDs

Theme	Key implementation challenges	Implementation strategies used
Designated space and operational support	Access to an appropriate assessment room within ED challenging given competing priorities	Audit and provide feedback* Involve executive boards (hospital management structures)*
Timely access to clinical input and supervision	Challenges in provision of timely consultant input and clinical supervision in some hospitals	Provide clinical supervision†
Navigating tensions in the ED through collaborative relationships	Parallel assessment of psychosocial and medical needs Compatibility of programme components with busy, fast-paced ED environment Limited ED staff awareness of self-harm and suicidal behaviours	Train-the-trainer strategies† Promote network weaving (between programme and ED staff)*
Nurse specialist training and networking	Increased autonomy in nurse specialist role and need for developing specialist skills Challenging crisis-nature of work	Use advisory boards and workgroups*† Conduct ongoing training/educational meetings† Promote network weaving (programme staff across sites)†
Varied availability of aftercare	Challenges in timely response of community providers for aftercare	Promote network weaving (with aftercare providers)*
Adaptability of programme delivery	Flexibility in organisation of work posing challenge in some hospitals where planning perceived as limited Varying hours covered by CNSs and out-of-hours cover by psychiatry trainees less familiar with programme	Conduct educational meetings*
Nationally led implementation strategies	Lack of clear mechanisms to mandate implementation at national level	National facilitation of strategies detailed above as well as Develop and organise quality monitoring systems†[Table-fn T2_FN3]

Note: implementation strategies are aligned with the Expert Recommendations for Implementing Change (ERIC).

* Strategies that emerged in response to implementation challenges.

† Strategies that were explicitly part of original model documentation and plans.

CNSs, clinical nurse specialists; ED, emergency department; NCPSHI, National Clinical Programme for Self-harm and Suicide-related Ideation.

## Discussion

This is the first study to examine factors affecting the implementation of a national programme to enhance care for people presenting to ED with self-harm or suicidal ideation drawing on experiences across hospital sites. Positive perceptions of the programme and the associated standardisation of care accompanied by nationally led implementation strategies were enablers of implementation. These findings extend our understanding of the previously reported positive clinical impact of the NCPSHI, with the greatest improvements in hospitals that did not have dedicated staff or clear approaches for managing self-harm or suicidal ideation prior to the introduction of the programme.^19^ However, similar to another National Clinical Programme in Ireland, the National Integrated Care Programme for Diabetes, our study identified variation in hospital resources and so implementation cannot be considered as a ‘one size fits all’ approach.^34^ While the care components were standardised, the ‘how’ of delivering the programme and organisation of work was not specified and were negotiated locally within each hospital, which had the potential to be helpful or harmful depending on the context. There was limited evidence of efforts to assess local context and needs prior to the implementation of the NCPSHI. For future programmes, conducting a local needs assessment, including local barriers and enablers, would allow for tailoring implementation to the needs of specific sites while clarifying core components of the programme.^32 33^

Collaboration with other ED care providers was reported to be of central importance to implementation. There were challenges of compatibility in delivering mental healthcare within the ED such as time pressures and limited appropriate space, as noted elsewhere.^22 23 35^ A large component of the CNS role involved communicating about the programme and gaining the support of other stakeholders in the ED and hospital, which was a challenge in some hospitals. Our study highlights the need to identify and support the skill development of CNSs as champions of implementation, aligned with other national programmes where champions were seen as key to introducing shifts in roles and work boundaries^34^ and sustaining implementation.^36^ Closer collaboration between liaison psychiatry and community providers has also been suggested as a strategy to address challenges in delayed access to aftercare^22 37^ noted in this study and elsewhere.^37 38^

CNS burnout and turnover were identified as challenges in our study due to work-related stress, work overload and career progression challenges. Addressing staff turnover is a key consideration for the sustainability of the NCPSHI.^39^ Availability of clinical support and supervision were identified as important to prevent and reduce burnout in this study, reflecting the broader literature.^40^ While clinical supervision was being provided in some hospitals, it was not consistent across all. In line with the literature on implementation strategies,^33^ provision of clinical supervision should involve training clinical supervisors and providing resources to ensure that clinical supervision occurs. Advisory boards/groups, developed to inform implementation of the NCPSHI, may be particularly useful in identifying and addressing barriers related to job satisfaction and retention.^33 41^

Examining implementation over time, barriers within the inner setting in the early years reduced as the programme moved into a sustainment phase such as the availability of a dedicated liaison consultant, availability of a designated assessment room and relationships with ED staff. These improvements could, at least in part, be attributed to the role of the NCPSHI national programme team engaging in strategies to support implementation over time. The strategies used by NCPSHI align with those most commonly used to support the sustainability of an intervention, namely to train and educate stakeholders, develop stakeholder interrelationships and evaluate and monitor programmes.^24 36^ The NCPSHI involved monitoring systems and audit,^42^ ongoing support and training of NCPSHI providers, and train-the-trainer approaches to increase collaboration with ED staff.

Organisational leadership and integration of interventions into existing systems have also been identified as predictors of sustainment.^24^ While the leadership from the national programme team was a critical factor in supporting the implementation of the NCPSHI, this study highlighted challenges around mechanisms for the implementation of such a programme within the health service and the lack of clarity around executive authority for implementation decisions, similar to other national programmes in Ireland.^43^ To further develop national programmes like the NCPSHI, it will be important to ensure integration and coherence between strategic and operational channels of the health service and to clarify mechanisms for implementation that flow between national and hospital level.

There are a number of strengths to this study. CFIR V.2.0 was used^25 26^ to guide data collection and analysis to ensure pertinent determinants were identified and to aid the comprehensibility and use of findings. Measures undertaken to enhance the credibility of the findings included independent coding of a sample of interviews (n=6, 20%) by a second researcher, discussion among the research team to encourage critical reflection on the potential impact of personal or research-based biases and presentation of the findings to the broader network of clinicians and managerial staff involved in delivering the programme. Nevertheless, this study also had some limitations. The retrospective nature of the study means that some recall challenges may have affected accounts of early experiences of implementation. We sought to address this limitation by recruiting participants who were involved in implementing the programme from the early years, along with structuring the interview guide to aid recollection over the full period of data collection. Our findings also represent the experiences of hospitals that were implementing the programme by 2015. Different factors may have affected implementation in hospitals that began implementation later. We were successful in ensuring representation across most key staff roles. However, we did not capture the perspectives of psychiatry trainees who have a key role in delivering the programme out-of-hours. This manuscript focused on identifying factors affecting the implementation of the programme overall. A follow-up study is in progress, the aim of which is to examine the implementation of each component of care.

## Conclusion

This study identified factors affecting the national implementation and sustainment of a programme to support people presenting with self-harm or suicide-related ideation to the ED, a key juncture to support this at-risk group. The perceived value of the programme introduced was an enabler of implementation along with oversight from a national team. Within the inner setting, early barriers in some hospitals included access to a designated assessment room, timely access to clinical input and collaboration with ED staff. Implementation strategies reduced barriers over time including ongoing training and education, developing stakeholder interrelationships and evaluation and monitoring. Continued efforts are needed to support nurse specialists delivering the programme and foster partnerships with community providers to improve the transition to aftercare.

## Supplementary material

10.1136/bmjqs-2024-017415 online supplemental file 1

10.1136/bmjqs-2024-017415 online supplemental file 2

## Data Availability

No data are available.
