# Supplementary material for: Factors affecting implementation of a National Clinical Programme for self-harm in hospital emergency departments: a qualitative study
Source: BMJ Qual Saf. 2024 Oct 8;34(10):e017415. doi: 10.1136/bmjqs-2024-017415 (PMC12505067; doi:10.1136/bmjqs-2024-017415)
Supplement: online supplemental file 1 [file bmjqs-34-10-s001.pdf]

Factors affecting implementation of a national clinical programme for self-harm in hospital emergency departments: a qualitative study.

Supplementary File 1

Interview Schedule – Clinical staff

Interviewer notes

- Explain that there will be two sections to the interview: firstly, to focus on early implementation of the programme, specifically the period 2015-2017 and secondly, to consider how the programme has been implemented in recent years from approximately 2019 [the year before the pandemic] onwards, including during the Covid-19 pandemic.
- The interviewer should note issues in the early phase implementation and follow-up to ask how these progressed in later/ongoing implementation (aka sustainment).
- The interviewer should seek to clarify the timeframes that the participant is describing, where needed, to differentiate issues in early implementation versus later implementation (aka sustainment).

Introduction

1. Name, role and length of time in post/working on NCP, years of experience.

Section 1: Early implementation (2015-2017)

2. How did your role come about? Can you tell me about your working role in the early days? [tailor this to situation i.e. newly recruited or liaison nurse taking on SH duties]
3. **Perceptions of the programme components:** There are a number of core components of the NCP including:
    a. biopsychosocial assessment,
    b. compassionate care,
    c. involvement of next kin,
    d. emergency care plan,
    e. follow-up and bridging to next care.

    What do you think of these components of care?

    E.g. the evidence underpinning the components; the extent to which the components are a better way of doing things than other practice; the complexity of the programme; the design of the programme and supporting materials.

    What was your experience of putting the components of care into practice in the early days?

    E.g. What was your experience of bridging to next care?

4. In your experience, what were some of the main challenges of putting the programme into practice in the early days?
5. Was it possible to resolve the challenges over time and if so, how?
6. What factors helped you or enabled you to implement the programme?

Factors affecting implementation of a national clinical programme for self-harm in hospital emergency departments: a qualitative study.

Prompts to probe further determinants of implementation (to support questions 4, 5, & 6)

a) **Team involved, relationships and communication:** Can you tell me about the main staff involved in delivering the programme in your hospital? How did you work together to deliver the programme? What did you think of the communication between staff involved in the programme?
b) **Hospital systems and structures:** Did you have to adapt the programme to fit in with the hospital systems/structures at the time? What kinds of changes were needed?
c) **Resources (e.g. materials, office, assessment room):** Did you have sufficient resources to implement the programme at the beginning? What additional resources were needed?
d) **Training and information needs:** Were you able to deliver training to other staff within the hospital, based on the train-the-trainer model. What was your experience of this? What additional training or educational resources, if any, would you have found helpful?
e) **Support from management:** How did hospital management support the programme? Can you give an example of an issue arising and what type of response you received? What steps, if any, were taken to encourage individuals to implement the programme?
f) **Acceptance by other staff:** How well was the programme accepted by other members of staff?
g) **Supervision:** What supervision/support did you have to carry out your role?
h) **Monitoring and feedback:** What was your experience of data input as part of the programme (if applicable)? In what ways has this data been used to inform your work in the hospital?
i) **Policies and guidelines:** Were there policies or guidelines in place that impacted the implementation of the programme?

Section 2: Later implementation (2019 onwards)

7. We have spoken a lot about the early stages of implementation. Could you tell me what the programme has been like since then? Have there been any changes in how it is delivered?
8. What are the main barriers that have affected the programme in recent years? Have you been able to address those barriers and if so, how? *[note what interviewee has raised in section 1]*
9. Has anything made implementation easier over the recent years?
10. How well do you think the programme in its current form meets the needs of the people who present to the ED with suicidal ideation or self-harm?
11. What do you think needs to happen to help the programme move forward? What, if anything, would you adapt going forward, either to the programme itself or how it is delivered?

Closing

12. What advice would you give to a team implementing the programme from day one?
13. Is there anything else you didn’t mention today that you would like to add?
