# Supplementary material for: Factors affecting implementation of a National Clinical Programme for self-harm in hospital emergency departments: a qualitative study
Source: BMJ Qual Saf. 2024 Oct 8;34(10):e017415. doi: 10.1136/bmjqs-2024-017415 (PMC12505067; doi:10.1136/bmjqs-2024-017415)
Supplement: online supplemental file 2 [file bmjqs-34-10-s002.pdf]

Factors affecting implementation of a national clinical programme for self-harm in hospital emergency departments: a qualitative study.

Supplementary File 2

Themes and influential CFIR constructs

Theme	Domains	Influential constructs
**National, standardised guidance for Emergency Departments (EDs)**	Innovation	- Innovation source<br>- Innovation relative advantage<br>- Innovation evidence-base<br>- Innovation design<br>- Innovation complexity
**Designated space and operational support**	Inner setting	- Available resources<br>- Relative priority<br>- Physical infrastructure<br>- Information technology infrastructure<br>- Relational connections
	Individuals	- Leaders [Nursing management]
**Timely access to clinical input and supervision**	Inner setting	- Tension for change<br>- Relational connections<br>- Communications<br>- Culture
	Individuals	- Leaders [Consultant leads]
**Navigating tensions in the ED through collaborative relationships**	Inner setting	- Relational connections<br>- Communications<br>- Tension for change<br>- Culture
	Individuals	- Innovation deliverers [ED staff]
**Nurse Specialists training and networking**	Individuals	- Capability<br>- Opportunity<br>- Motivation
	Inner setting	- Access to knowledge & information
	Implementation process	- Tailoring strategies
**Varied availability of aftercare**	Outer setting	- Local conditions<br>- Partnerships & connections<br>- Local attitudes
**Adaptability of programme delivery**	Inner setting	- Work infrastructure<br>- Compatibility
	Implementation process	- Teaming<br>- Engaging innovation deliverers<br>- Planning
	Individuals	- Innovation deliverers [Nurse Specialists & Psychiatry Trainees]
**Nationally led implementation strategies**	Outer setting	- Financing<br>- Policies & laws<br>- Partnerships & connections<br>- External pressure<br>- National-level oversight *[inductive code added to CFIR]*
	Implementation process	- Reflecting and evaluating<br>- Tailoring strategies
	Individuals	- Other implementation support [National programme team]
